# On Modern Methods of Diagnosis in Gastric Affections

**Published:** 1891-06

**Authors:** J. Michell Clarke

**Affiliations:** Assistant-Physician and Pathologist, Bristol General Hospital; Assistant-Lecturer on Physiology, Bristol Medical School


THE BRISTOL

Medico-Chirurgical Journal.

JUNE, 1891.

### ON MODERN METHODS OF DIAGNOSIS IN GASTRIC AFFECTIONS.

BY

J. Michell Clarke, M.A., M.B., M.R.C.P.,

*Assistant-Physician and Pathologist, Bristol General Hospital; Assistant-Lecturer on Physiology, Bristol Medical School.*

Of late years many new procedures have been introduced with the object of improving our methods of diagnosis in gastric affections by direct analysis of the gastric juice, and of thus enabling us by the light of more exact knowledge of the morbid process to interfere with greater precision and success. The methods employed—which, roughly speaking, consist in the withdrawal of a portion of the stomach-contents during various stages of digestion, without risk or undue discomfort to the patient, and subsequent analysis of them by special tests—have already materially added to our knowledge of the processes that normally take place in gastric digestion and of their pathological derangements. To be useful in ordinary clinical work, the tests employed must be capable of easy application, and be at the same time fairly accurate. The object of the present paper is, to show that by the selection and application of the more important and trustworthy of the tests devised valuable aid in diagnosis and treatment may be obtained without the expenditure of much time or labour, and that these tests may be carried out in ordinary out-patient work. Under such conditions the more elaborate procedures are hardly possible.

By the labours of Ewald, Boas, von Jaksch, Mathieu, and others, it has been conclusively determined that hydrochloric acid is the essential acid of the gastric juice. It is not, however, secreted at once on the ingestion of food; for at the commencement of digestion, during a period which varies between twenty and fifty minutes, lactic acid is the only acid present, probably derived partly from the meat and partly from the carbohydrates of the food. Then hydrochloric acid appears, and both acids are present together for a time, the lactic acid disappearing at from sixty to ninety minutes after ingestion of food. Hydrochloric should be the only acid present at from ninety minutes to two hours, and digestion is then most actively proceeding. It disappears when the stomach contents pass into the duodenum, and is not found in the stomach in the intervals between meals. Moreover, its secretion varies with the kind of food ingested: the presence of carbohydrates in excess retards its appearance; on a mixed diet it should be found at the end of from thirty to fifty minutes after a meal, and after a meal of cooked albumen only, *e.g.* white of egg, it sometimes appears as early as fifteen minutes. In every case hydrochloric acid should be the only one present at the end of ninety minutes, and the activity of the gastric juice is in close proportion to the amount of it secreted. The method of procedure which I have found capable of being carried out in a reasonably short time, and from which we may obtain a fair knowledge of the events taking place during gastric digestion, is the following:

The patient should take a "test-meal," which may consist of a little weak tea and bread and butter, with or without an egg. Milk is not to be recommended, as it has the property of forming compounds with hydrochloric acid. A soft India-rubber nasal tube is then passed, either through the mouth or through the nose, into the stomach. With the palm of the hand pressure is made over the patient's stomach, and he is directed to cough. It is most convenient that he should be lying down, with his head turned to one side. After coughing a few times, a little of the gastric juice flows out of the end of the tube, and is collected in a flask. In cases where there is vomiting after meals, the vomit may sometimes be used for analysis. The fluid thus obtained is filtered; the deposit examined under the microscope, and the acidity of the filtrate determined. Prof. v. Jaksch thinks it is better to use the unfiltered gastric contents, as he found some loss of acidity took place during filtration. To determine the acidity, 10 c.cc. of the filtered gastric contents are taken; two or three drops of phenol-phthalein, which strikes a lively red colour in the presence of free alkali, added; and then a deci-normal solution of sodium hydrate run in from a burette. 1 c.c. of the latter neutralises .00364 gramme pure hydrochloric acid. Whilst the deci-normal solution is added the fluid must be constantly stirred, and when the red colour appears the number of c.cc. of the deci-normal solution used is read off, and the total acidity thus calculated in terms of hydrochloric acid. Next we test for hydrochloric, lactic, and butyric acids.

The presence of hydrochloric acid can be determined by several colour reactions. Those that seem most reliable, and the only ones with which I am personally acquainted, are Günzburg's phloro-glucin test, the tropæolin, Congo-red, and benzo-purpurin 6 B tests. Of these, I no longer use tropæolin, as it is not so delicate as the others. Test-papers may be made by soaking strips of filter-paper in saturated solutions of Congo-red and benzo-purpurin 6 B. Hydrochloric acid gives a blackish-blue, or blue, colouration with Congo-red; it is open to the fallacy that the organic acids, if present in any quantity, give also a deep brownish-black colouration, difficult to distinguish from that due to hydrochloric acid; but, so far as I have observed, this has never the blue tinge of the latter reaction. With benzo-purpurin 6 B, hydrochloric acid gives a dark or light violet colour, according to the amount present. The organic acids here also give a brownish black colour, which, however, may be dispelled by shaking up the paper in a test-tube with sulphuric ether (which must not itself be acid), whereas the colouration due to hydrochloric acid is unaffected. The presence of peptone, serum-albumin, or acid salts is said not to interfere with this reaction, while they certainly do hinder that of Congo-red. Günzburg's reagent is composed of 2 grms. phloro-glucin and 1 grm. vanillin in 100 parts absolute alcohol. In using it, a few drops of the gastric juice and an equal quantity of the reagent are put in a porcelain dish, and gently heated over a spirit-lamp. As evaporation takes place, a beautiful rosy-red colour appears on the dish. Care must be taken that overheating or charring of the mixture does not occur, as in that case the reaction does not appear. This test is very delicate, giving the reaction with $\frac{1}{20}$ hydrochloric acid in 1000 parts, and seems the most trustworthy if a little care is exercised in carrying it out; the reaction is not interfered with by the presence of peptone, albumin, or acid salts. Inorganic salts and chloride of calcium, 2 per cent., will give the same reaction, but are never present in sufficient quantities in the gastric juice. The only practical source of error, according to Günzburg is, that very rarely lactate or acetate of soda may be present in sufficient quantity to lead to confusion.

Before testing for hydrochloric, the lactic acid present may be removed by agitating the gastric juice with ether, subsequently decanting off the ether, evaporating it, and dissolving the residue in water. Uffelmann's reagent is a distinguishing test; it must be freshly made by adding one drop of liq. ferri perchloridi to a mixture of 10 c.cc. of a 4 per cent. solution of carbolic acid and 20 c.cc. of distilled water. This gives an amethyst-blue liquid, which is bleached on the addition of hydrochloric, turned yellow by lactic, and ashen-grey by butyric acid. Butyric acid is also detected by its characteristic odour.

The question further comes in as to whether the acidity is due mainly to free acid, to acid salts, or to acids in organic combination. Leo has devised a means of determining whether the acidity is due to free acid or to acid salts, which depends upon the fact that dry calcium carbonate fully neutralizes free acids, but does not decompose acid phosphates. The method is thus described by Prof. D. J. Hamilton: "A little of the liquid, a few drops, is placed in a watch-glass; a pinch of powdered calcium carbonate is added, and mixed with a glass rod. The reaction is then taken with blue litmus paper, and compared with that of the original liquid. If the litmus is not coloured red, we have to do with free acid only; if the reddening of the litmus has become less intense than in the original liquid, free acids and acid salts have been simultaneously present; and if the reaction does not change, acid salts have been the exclusive source of the acidity."[1] Lastly, the gastric contents may be tested for the presence of peptones, propeptone, albumin, and sugar. Small cubes of boiled white of egg may be placed in the gastric juice, and kept at the body temperature, in order to test the digestive power; if it is found to be deficient, pepsin, hydrochloric acid, or pepsin and hydrochloric acid, may be added to fresh portions with white of egg, and the digestive agent in default thus determined. The quantitative estimation of hydrochloric acid is too long and difficult a process to be useful for ordinary clinical work; rough quantitative estimations according to the degree of colouration with the above reagents have been devised, but are not sufficiently accurate to be of value. With a little practice the above tests, though they seem long when described, can be carried out in a reasonable time. The whole process may be summed up thus:—

(1) A portion of the gastric contents is removed two hours after a meal, and filtered.

(2) The total acidity of the filtrate is estimated, and it is determined whether the acidity is due to free acid, acid salts, or both combined.

[1] *British Medical Journal*, Vol. II., 1890, p. 323.

(3) The presence of hydrochloric acid is shown by benzo-purpurin and Günzburg's reagent.

(4) Lactic and butyric acids tested for by Uffelmann's reagent, by odour, &c.

(5) Determination of digestive potency of the gastric juice.

Mathieu[1] has recently made some interesting observations on the determination of the total acidity of the gastric contents by means of phenol-phthalein, and by another body tournesol, which also shows a change of colour in presence of free alkali. Phenol-phthalein always shows an excess of acidity over tournesol, due to the fact that peptone, acid albumin, and leucin act upon it, whilst they do not affect the latter. Organic acids also show the same difference, and this difference between the two acidities is augmented in direct proportion to the total acidity. He concludes that the difference in acidity is not due to azotised bodies alone, and that the organic acids not only exist in free state, but also in combination. Hayem and Winter (quoted by Mathieu), from the result of their investigations upon the gastric contents, are of opinion that the changes produced in the food by the gastric juice are preparatory to those that take place in the intestine, rather than of intrinsic importance in themselves; that the stomach restrains and moderates fermentative processes; and that the quantity of peptone produced is of secondary importance. Whilst investigating the chemical changes, we must not forget the very important part played by the motor functions of the stomach, and we should complete our studies by determining the time after a meal at which the viscus

[1] *Gaz. des hôpitaux*, February 17th, 1891.

empties itself. This should take place within five or six hours after a meal. There are variations within the limits of health, but longer than six hours is probably always pathological; fermentative changes, with the development of lactic and butyric acids, then take place, and after seven hours these are the only changes that occur.[1] In cases of undue retention the stomach undergoes dilatation, and this can be satisfactorily determined by palpation, which gives a succussion splash over the stomach-area, and its limits can with a little care and practice be thus accurately defined. If the lower border of the stomach thus determined passes beyond a line drawn from the umbilicus to the salient angle of the left false ribs in the intervals between meals, or if at any time after a meal it passes below this line, a pathological degree of distension is present. At the same time the area of stomach-resonance over the lower ribs may extend upwards beyond the sixth rib, and to the left beyond the normal limits.

Or we may make use of salol (Ewald's test) to determine the time at which the food passes out of the stomach. This body is unchanged in the stomach, but broken up in the duodenum into phenol and salicylic acid; the latter at once passes into the urine, where it gives the characteristic deep-purple colour with perchloride of iron. Ewald thought that the time of its first appearance in the urine gave certain information as to the motor power of the gastric muscle; but this time has been shown to be very variable, and the test must be used in another way. According to Huber, the reaction should normally *disappear* from the urine twenty-six hours after ingestion, while in cases of impaired

[1] Bouchard, *Sur les Auto-intoxications*, Chap. xix.

mobility it may still be obtained after thirty to thirty-six hours.

Rossbach[1] has recently shown that in dogs the contents of the stomach are not ejected from time to time as digestion proceeds, as is generally stated in the text books, but that the pylorus remains firmly closed until from four to six hours have elapsed and then relaxes, and the stomach-contents are ejected in four or five successive squirts; active contractions of the pyloric end of the stomach playing an important part in the process.

The most important facts learnt from the above methods of clinical investigation are as follows: The pathological variations of acidity may be divided into two groups—excess and deficiency of hydrochloric acid.

First, hydrochloric acid may be present in excess only during digestion; the symptoms of dyspepsia coming on in from thirty to sixty minutes after a meal, and consisting of acid regurgitations and epigastric pain.

Secondly, hydrochloric acid may be present in excess both during meals and in the intervals between them. In this case there is generally dilatation of the stomach, which Riegel thinks may arise from spasm of the pylorus, excited by the excessive acidity, hindering the outflow of the stomach-contents. The patients are anæmic and often emaciated; they suffer much from epigastric pains, flatulence, and sometimes from vomiting; especially characteristic are nocturnal attacks of severe pain, and often vomiting. The conditions may thus closely resemble cancer of the stomach.

According to Mathieu[2] the digestion of meat is

[1] *Deutsch. Archiv. f. klin. Med.*, 1890, Bd. xlvi. 276. Abstract *Int. Journ. Med. Sci.*, February, 1891.

[2] *Gaz. des hôpitaux*, February and April, 1888.

complete, of carbohydrates *nil*, in these cases: he states that the exaggerated secretion of hydrochloric acid may come on in "crises," with pain in the epigastrium, acid vomitings, and severe headache, and suggests that the "gastric crises" of tabes dorsalis are due to this affection of the stomach.

In a case of this acid dyspepsia with slight dilatation we have found the total acidity to be .4 per cent., instead of .1 or .2 per cent.

Thirdly, in round ulcer the quantity of hydrochloric acid is generally, but not invariably, increased: in a patient now under treatment, the total acidity is .3 per cent. In the acid dyspepsia of chlorosis, it is common to find some excess of hydrochloric acid in the gastric contents. These facts are significant when we remember that round ulcer is especially liable to occur in anæmic girls; possibly the excessive secretion of hydrochloric acid is the antecedent stage, and in the bloodless condition of the stomach-walls leads on to the formation of ulcer.

Besides hydrochloric, butyric and acetic acids may be in excess; but when this is the case, the amount of hydrochloric is generally deficient. The secretion of hydrochloric acid is deficient or absent in (1) cancer of the stomach, (2) atrophy of the mucous membrane, (3) sometimes in the continued fevers, (4) in dyspepsia nervosa, and (5) very occasionally in persons otherwise in apparent good health. The most frequent and important cases in which hydrochloric acid is absent from the stomach during digestion are those of cancer, and this fact often affords us valuable aid in diagnosis. Atrophy of the gastric mucous membrane is a rare condition; the diagnosis between cancer of the stomach and fever will rarely be a matter of difficulty; there remain the cases where hydrochloric acid is absent from the gastric juice in apparent health. Hayem and Winter mention six cases of the latter class. One of this kind has recently come under my notice. The patient was a lad of sixteen, who complained that about an hour after meals he suffered from eructations of fluid which tasted and smelt like rotten eggs. He had no pain or vomiting, and seemed in good health. On examining the gastric juice, it gave only a very feeble acid reaction, and with the smallest possible trace of hydrochloric acid.

If the possible exceptions to the rule that the secretion of hydrochloric acid is absent in cases of cancer of the stomach be borne in mind, I do not think that they militate seriously against the value of this reaction as an aid to diagnosis in cases where the existence of cancer is uncertain, but seems probable. If hydrochloric acid is found absent after repeated examination at various stages of the digestive process, the presumption in favour of cancer is very strong. In eight cases of cancer of the stomach in which I examined the gastric contents on several occasions, in each case hydrochloric acid was uniformly absent. The following two cases illustrate the manner in which the absence of reaction was of great aid in diagnosis:

The first, a man of 77, fairly well nourished and of healthy appearance, had suffered for three months from epigastric pain and sickness after meals, and on one occasion from slight hæmatemesis. The epigastrium was tender; but it was difficult to explore it thoroughly, from the roundness of the thoracic cage, and the prominence of the lower ribs. We could never discover any tumour, nor was there any dulness over the stomach, which, moreover, was at no time dilated. Hydrochloric acid was absent from the gastric juice. The man emaciated, grew slowly worse, and died in about two months. At the autopsy a cancerous growth was found at the cardiac end of the stomach, close to the entrance of the œsophagus, and, therefore, so placed that it could never have been felt during life.

The second patient, a man of 60 years of age, had for more than six months suffered from pains after food, and acid eructations; very occasionally he had vomited, but had never brought up blood. The stomach was moderately dilated, and repeated examination failed to show the presence of hydrochloric acid in the gastric contents. The pain and sickness were relieved by daily washing-out of the stomach with warm solution of boracic acid. He did not, however, entirely lose these symptoms, and recently dulness was detected over the right side of the epigastrium, where a small hard nodule can now be felt, undoubtedly of cancerous nature.

Whether hydrochloric acid is absent in the early stages of cancer of the stomach, I am unable to state from personal observation; that it is not necessary for the stomach-walls to be extensively infiltrated with new growth, the following case shows:

Sarah T., æt. 47, married, of healthy family; no children; no previous illness. She came under my care in September, 1890, and stated that the present illness began in January, 1890, with pains in hands and head. The pains after a time shifted to the left hypochondriac region, where they have since remained; she lost her appetite entirely, and became very weak. The pain was worse after meals; she was losing flesh, and suffered much from constipation. There had been no vomiting. The patient was anæmic and emaciated. On examination the first sound of the heart was reduplicated at the apex; but otherwise the thoracic organs were healthy. The tongue was furred; the abdomen rather hollow; and on the right side, about two inches above and a little to the right of the umbilicus, was a small irregular and intensely hard lump, about two inches in its longest diameter, which was from above downwards. It was intensely tender, and moved freely with respiration. The stomach was not dilated, and there was no ascites. The gastric contents at no time contained hydrochloric, but a good deal of lactic and butyric acids. She died in about a fortnight; and for a few days before her death the stomach was enormously dilated, and she suffered much from vomiting. At the autopsy, a small nodule, of almost cartilaginous hardness was found at the pylorus, constricting the orifice; microscopic examination showed it to be a scirrhus growth. This case only came under observation very late in the disease, but it shows that the new growth may be very small and entirely limited to the pylorus, and yet hydrochloric acid may fail to be secreted. It is very difficult to see why such a limited growth should, even in the late stages, interfere with the secretion of hydrochloric acid; at an earlier stage it would probably not do so.

The diagnosis of gastric carcinoma is often a matter of great difficulty, and especially so in cases where there is dilatation of the stomach. This dilatation may arise from organic disease obstructing in some way the pyloric opening; or, on the other hand, it may be "simple"—that is to say, not due to organic disease, but from a functional weakness of the gastric walls. This latter is not an uncommon condition in both men and women, and arises in many conditions, for instance, in persons who overload the stomach by too heavy and too frequent meals, in the course of the continued fevers or during convalescence from acute diseases, in alcoholism, neurasthenia, phthisis, severe anæmia, chronic rheumatoid arthritis, and other debilitated states. Bouchard believes that there is an hereditary weakness of the stomach-walls in some cases. If we are, however, aware of its occurrence under such circumstances, the diagnosis can generally be made with certainty. It is, however, in conditions where dilatation exists together with a tumour of some kind in the neighbourhood of the stomach that real difficulty arises. The cases that most nearly simulate cancer of the stomach are those of cancer of the head of the pancreas and cancer of the gall-bladder, either of which may compress the pylorus and so cause dilatation of the stomach. The same thing may more rarely happen in cases of new growth in the left lobe of the liver; and, lastly, may be produced by the thickening and contraction around an ulcer seated close to the pylorus, either in the stomach or in the duodenum. In these cases of ulcer, the diagnosis from ordinary physical signs and symptoms alone may be impossible, especially when the patient is in late middle-life. Duodenal ulcer in this situation occurs most commonly after the age of thirty, and may be present in elderly people. At an autopsy recently, I found a simple ulcer of the duodenum in a man of sixty years of age, which was surrounded by a mass of inflammatory new formation, causing some obstruction of the pylorus with dilatation of the stomach, but apparently not sufficient to give rise to symptoms during life more urgent than those of a moderate degree of dyspepsia.

The following is a good instance of the difficulties of distinction between gastric ulcer in the region of the pylorus, and cancer in the same situation:

William B., a labourer, æt. 45, had attended as an out-patient several times during the previous two years, for severe pains in the stomach, and vomiting after meals. The pain was always localised over the right side of the epigastrium, came on about an hour after eating, and was to some extent relieved when the meal was vomited. There had never been any hæmatemesis. Beyond severe dyspepsia, for many years he had had no bad illness, and had always been temperate. On July 8th, 1887, he came to me with an aggravation of the pain and vomiting, and on examination there was then found dulness over the right epigastric region, which was fuller than the left, and on palpation in this situation an ill-defined rather hard tumour, about the size of a large orange and excessively tender was felt. The stomach was moderately dilated. On milk diet, with the administration of opium and bismuth, the pain was partly relieved; but on September 1st he was taken suddenly ill with intense pain in the stomach, and for the first time vomited a large quantity of blood. He died in a state of collapse some twelve hours later. At the autopsy acute general peritonitis was present. The stomach at the pyloric end was the seat of a tumour, about the size of the two fists, composed of firm, hard, pale tissue. Two large deep ulcers, with ragged walls, were found in this tumour, and at the base of one of these perforation into the peritoneal cavity had occurred, and hemorrhage had taken place from it. The surrounding organs were matted together by adhesions, the pylorus was very much narrowed, and the stomach dilated. The tumour appeared to consist of inflammatory new formation thrown out around the ulcers, and careful microscopical examination of portions from several parts of the tumour showed that this was its structure.

If, as seems at present to be the case, the amount of hydrochloric acid secreted is generally increased and always present in cases of ulcer, and is absent in carcinoma, we have in this test a most valuable diagnostic agent in cases such as the above, where the distinction on other grounds is exceedingly difficult; and it will similarly be of service in those cases of cancer of the head of the pancreas or of other viscera where new growth in the stomach is closely simulated. I am not contending that in all patients with dyspepsia, or in whom dyspeptic symptoms occur, a portion of the gastric contents should be removed and analysed according to the method detailed above; but I think that it should be done where new growth or ulcer is suspected, and that when dyspepsia does not yield to treatment we may often obtain valuable information from the procedure as to what ingredient of the gastric juice is deficient or in excess, and thus be enabled to modify the treatment accordingly, and so give relief. In conclusion, it may be well to add that by using a soft India-rubber tube without a stiffened end—such as that sold under the name of Jaques' patent, which answers the purpose perfectly well—there is no risk of doing harm even in cases of ulcer.

